# Oxidative Stress-Induced Ferroptosis in Cardiovascular Diseases and Epigenetic Mechanisms

**DOI:** 10.3389/fcell.2021.685775

**Published:** 2021-08-19

**Authors:** Jiamin Li, Yunxiang Zhou, Hui Wang, Jianyao Lou, Cameron Lenahan, Shiqi Gao, Xiaoyu Wang, Yongchuan Deng, Han Chen, Anwen Shao

**Affiliations:** ^1^Department of Cardiology, Zhejiang Provincial Key Lab of Cardiovascular Disease Diagnosis and Treatment, Second Affiliated Hospital, Zhejiang University School of Medicine, Hangzhou, China; ^2^Department of Surgical Oncology, The Second Affiliated Hospital, School of Medicine, Zhejiang University, Hangzhou, China; ^3^Department of Medical Oncology, The Second Affiliated Hospital, School of Medicine, Zhejiang University, Hangzhou, China; ^4^Department of General Surgery, The Second Affiliated Hospital, School of Medicine, Zhejiang University, Hangzhou, China; ^5^Burrell College of Osteopathic Medicine, Las Cruces, NM, United States; ^6^Center for Neuroscience Research, School of Medicine, Loma Linda University, Loma Linda, CA, United States; ^7^Department of Neurosurgery, The Second Affiliated Hospital, School of Medicine, Zhejiang University, Hangzhou, China

**Keywords:** iron, ferroptosis, oxidative stress, epigenetic regulators, cardiovascular diseases

## Abstract

The recently discovered ferroptosis is a new kind of iron-regulated cell death that differs from apoptosis and necrosis. Ferroptosis can be induced by an oxidative stress response, a crucial pathological process implicated in cardiovascular diseases (CVDs). Accordingly, mounting evidence shows that oxidative stress-induced ferroptosis plays a pivotal role in angio-cardiopathy. To date, the inhibitors and activators of ferroptosis, as well as the many involved signaling pathways, have been widely explored. Among which, epigenetic regulators, molecules that modify the package of DNA without altering the genome, emerge as a highly targeted, effective option to modify the signaling pathway of ferroptosis and oxidative stress, representing a novel and promising therapeutic potential target for CVDs. In this review, we will briefly summarize the mechanisms of ferroptosis, as well as the role that ferroptosis plays in various CVDs. We will also expound the epigenetic regulators of oxidative stress-induced ferroptosis, and the promise that these molecules hold for treating the intractable CVDs.

## Introduction

Cardiovascular diseases (CVDs), including heart conditions and vascular disorders, are the leading cause of mortality around the world, and comprise approximately one-third of annual deaths [World Health Organization (WHO), 2017]. Moreover, CVDs carry an enormous economic burden to every country, especially China and India [The Institute for Health Metrics and Evaluation (IHME), 2018]. The most prevalent CVDs include hypertension, coronary heart disease, atrial fibrillation, and valvular heart disease, but most CVDs develop into heart failure at the advanced or terminal stages. In 2017, update of the guidelines for the management of heart failure released by ACC/AHA/HFSA (American College of Cardiology Foundation/American Heart Association/Heart Failure Society of America), angiotensin receptor–neprilysin inhibitors (ARNI) (sacubitril/valsartan) and sinoatrial node modulators (ivabradine) were classified as the therapy for stage C heart failure in the evidence of level B-R, but require further high-quality randomized clinical trials to be conducted (Dixon et al., 2012; Yancy et al., 2017). The current treatment of CVDs is unsatisfactory, and the underlying mechanisms are not fully understood. As such, it is imperative that new mechanisms and corresponding therapeutic targets are explored.

Ferroptosis was first introduced by Dixon et al. (2012), and was featured as an iron-dependent lipid peroxidation, a regulated cell death that is different from apoptosis and necrosis. Currently, ferroptosis was defined as a unique iron-dependent form of non-apoptotic cell death triggered by erastin, an oncogenic RAS-selective lethal small molecule, and inhibited by ferrostatin-1 in cancer cells or glutamate-induced cell death in organotypic rat brain slices (Dixon et al., 2012). Mitochondria are crucial in ferroptosis, tricarboxylic acid (TCA) cycle participates in cysteine-deprivation induced ferroptosis and that the electron transport chain (ETC) regulates the process (Gao et al., 2019). Mitochondria participate in metabolism, and are the main source of reactive oxygen species (ROS) (Tang D. et al., 2021). Oxidative stress occurs when the antioxidant defense systems, such as GSH, coenzyme Q10, and tetrahydrobiopterin (BH4), cannot find equilibrium of ROS (Dixon et al., 2012; Bersuker et al., 2019; Doll et al., 2019; Kraft et al., 2020; Soula et al., 2020). GSH, the major antioxidant in mammalian cells, is tightly tuned intracellularly and extracellularly for homeostasis (Gao et al., 2018), and is also the key component in ferroptosis. Ferroptosis has been found in many diseases, such as cancer, CVD, neurological disease, and ischemia/reperfusion injuries in kidney, liver, lung, and skeletal muscle (Stamenkovic et al., 2019). Ferroptosis may be a potential mechanism underlying CVDs as many studies pointed out that ferroptosis have been implicated in CVDs (Birnbaum et al., 1996; Shiomi et al., 2004; Dabkowski et al., 2008; Fang et al., 2019).

The biological processes are regulated by genetics and epigenetics. Epigenetics is known as the unchanged nucleotide sequence of the gene that is modulated by several environmental factors while genetics irreversibly change the gene code via mutation (Borrelli et al., 2008). Epigenetics act on DNA or chromatin by DNA methylation, histone modifications, chromatin remodeling and noncoding RNAs (Prasher et al., 2020; Wu et al., 2020). Based on epidemiological studies, alteration of lifestyle and environment can reduce the risk of developing CVDs (Wang et al., 2013). It has been suggested that ferroptosis can be regulated by epigenetic, transcriptional, and post-translational mechanisms (Chen et al., 2020). Accumulating evidence indicates that a series of epigenetic regulators are involved in the processes of ferroptosis. In the present review, we will elaborate on the mechanism of ferroptosis, the roles of ferroptosis in CVDs, as well as the roles of epigenetic regulators in oxidative stress-induced ferroptosis, and we will offer an option for the therapeutic application of ferroptosis in CVDs.

## Mechanism of Ferroptosis

Cell death is frequently required to maintain the normal functions of the body/system, either under physiological conditions or pathophysiological circumstances. Two major classifications of cell death are apoptosis and necrosis. Other patterns of “non-classical” cell death, such as autophagy, pyroptosis, and necroptosis, reportedly also have important roles in cell survival and body function (Dixon et al., 2012).

Dolma et al. (2003) found a novel compound that can kill tumor cells without damaging isogenic normal cell counterparts. They named it “erastin,” and it induces nonapoptotic cell death in a RAS^V12^- and small T(ST)-dependent manner (Dolma et al., 2003). Furthermore, Yang and Stockwell (2008) found that two small molecules, RSL (ras-selective-lethal compound) 3 and RSL5, were lethal to tumors with oncogenic RAS, similar to the function of erastin. RSL3- or RSL5-induced cell death is considered iron-dependent as it could be inhibited by either iron chelation or decreased iron uptake, with increased levels of ROS (Yang and Stockwell, 2008). Ferroptotic cells cannot be restrained by inhibitors of necrosis, apoptosis, or autophagy and exhibit morphological changes in mitochondria, such as decreased size, increased membrane density, and reduction or disappearance of cristae (Xie et al., 2016). In 2012, the team of Dixon SJ conducted further research to support and extend this newly discovered form of regulated cell death. They proposed the concept of ferroptosis for the first time, and defined it as the regulatory cell death induced by the accumulation of lipid peroxides and ROS, which can be inhibited by lipid peroxide inhibitors and iron chelators (Dixon et al., 2012). Outer mitochondrial membrane (OMM) rupture was observed in immortalized fibroblasts and glutathione peroxidase 4 (GPX4)-inactivated kidney tissue (Angeli et al., 2014).

Iron is an important essential microelement in the human body, and plays a key role in maintaining homeostasis of the internal environment, and ensuring the normal physiological functions of cells. Iron in the human body is mostly distributed in the hemoglobin of red blood cells and the myoglobin of muscles, but a small amount exists in enzymes, such as cytochrome oxidase, peroxidase, and catalase. There are two types of iron ions: ferrous and ferric. Ferric ions bind to transferrin, and are transported into the cell, entering via the transferrin receptor 1 (TFR1) on the cell membrane (Gao M. et al., 2015). Ferrous ions reduced to ferric ions in the cell, and are then transported and released into the cytoplasmic iron pool. Ferrous ion can react with oxygen, and generates ROS, such as hydroxyl radical (•OH) and hydrogen peroxide (H_2_O_2_), in a process known as the Fenton reaction. Iron overload leads to an increase of ROS, which cause harm to DNA, protein, and lipids. The Haber–Weiss reaction provides •OH from the substrates of H_2_O_2_ and superoxide (•O_2_^–^): (1) Fe^3+^+•O_2_^–^→Fe^2+^+O_2_; (2) Fe^2+^+H_2_O_2_→Fe^3+^+OH^–^+•OH (Fenton reaction); (3) •O2^–^+H_2_O_2_→•OH+OH^–^+O_2_ (Gao M. et al., 2015). Cellular iron overload can impair mitochondrial oxidative phosphorylation and produce a large amount of ROS, even exceeding the scavenging ability of the body’s antioxidant system [e.g., glutathione (GSH) and GPX4], thereby oxidizing cell membranes, as well as the unsaturated fatty acids on cell and organelle membranes, forming lipid peroxides, destroying cell structure and function, and causing cell damage or death (Dixon et al., 2012; Ooko et al., 2015; Hassan et al., 2016).

**FIGURE 1:**
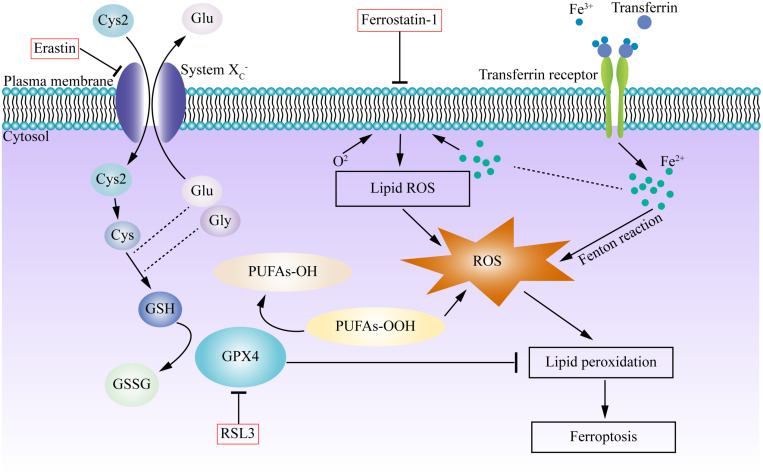
Graphic mechanisms of ferroptosis. Dysregulation of intracellular iron metabolism or glutathione peroxidation pathways leads to accumulation of lipid ROS and eventually causes ferroptosis. Various inducers and inhibitors are shown in the red box. Arrows indicate promotion; blunt-ended lines indicate inhibition. Excessive free irons are the foundation for ferroptosis execution. Circulated Fe^3+^ was combined with transferrin, and then entered into cells by transferrin receptor. Iron in Fe^3+^ form was deoxidized into iron in Fe^2+^, the latter generated ROS by Fenton reaction. System Xc- is an antiporter that imports cystine and exports glutamate, providing cysteine for glutathione synthesis, can be inhibited by erastin. GPX4 is a redox enzyme which reduces reactive aldehydes (PUFAs-OOH) to their alcohol form (PUFAs-OH), reduces ROS accumulation, plays important roles in the regulation of ferroptosis. RSL3 directly inhibit GPX4, which inhibits lipid peroxidation and prevent cell death, triggering the accumulation of ROS and boost ferroptosis. Ferrostatin-1 reduced the production of lipid ROS and attenuated ferroptosis. Cys, cysteine; Cys2, cystine; Glu, glutamate; Gly, glycine; GSH, glutathione; GSSH, glutathione disulfide; GPX4, glutathione peroxidase 4; PUFA, polyunsaturated fatty acid.

It is widely accepted that ferroptosis is regulated by the cystine/glutamate antiporter system (system Xc^–^) and GPX) (Dixon et al., 2012). System Xc^–^ is an amino acid antiporter, which mainly includes SLC7A11 (solute carrier family 7 member 11) and SLC3A2 (solute carrier family 3 member 2), which causes the exchange of cysteine and glutamate into and out of the cell, respectively, at a 1:1 ratio (Lewerenz et al., 2013). Glutathione is an important antioxidant and free radical scavenger in vivo, and can be categorized as either reduced (GSH) or oxidized (GSSG). GPx4 converts GSH to GSSG, GSH/GSSG constitutes an antioxidant system and provides reducing equivalents to eliminate oxidative species (Xie et al., 2016; Yang W. S. et al., 2014). The synthesis of GSH depends on the cysteine, which is made by cystine, and glutamate-cysteine ligase (GCL). As a member of the glutathione peroxidase family, GPX4 inhibits ferroptosis by decreasing the level of lipid peroxides (Liang et al., 2009). While erastin and RSL-3 are both inducers of ferroptosis, erastin depends on VDAC2/VDAC3 or downregulation of GSH. However, RSL-3 does not require the above-mentioned molecules. Lipid oxidation is observed in both erastin and RSL3-induced cell death. Further investigation verified that GPX4 is the target of RSL-3 through a binding site (Yang W. S. et al., 2014). Many inducers (e.g., erastin, RSL3, RSL5, buthioninesulfoximine, acetaminophen, fin, lanperisone, sulfasalazine, sorafenib, and artesunate) and inhibitors (e.g., ferrostatin, liproxstatin-1, and zileuton) of ferroptosis have been identified, but the specific mechanisms and pathways are diverse (Xie et al., 2016). In summary, ferroptosis is a complex process, and more pathways will be discussed in the following sections (Figure 1).

## Roles of Ferroptosis in CVDs

### The Pathophysiologic Mechanisms of CVDs

The heart is one of the most important organs, and is responsible for pumping blood throughout the body, providing energy and oxygen to nourish tissues and organs, and removing metabolites, such as carbon dioxide. There are complex regulatory mechanisms involved in maintaining normal cardiac function.

Inflammation is an important molecular trigger in CVD. Considerable evidence has shown the close relationship between inflammation and atherosclerosis (Ross, 1999; Libby and Ridker, 2006; Wong et al., 2012), and some anti-inflammatory drugs, such as statins, work to prevent cardiovascular events (Shepherd et al., 1995; Ridker et al., 2001, 2005).

Endothelial dysfunction arises when endothelial cells (EC) are injured, or if there is an imbalance between vasoconstriction and vasodilation (Chatzizisis et al., 2007). The gathering of low-density lipoprotein (LDL) in the subendothelial layer is thought to be the initial event of atherogenesis (Russell et al., 1989; Williams and Tabas, 1995; Tabas et al., 2007). Oxidative-LDLs (Ox-LDLs) induce proinflammatory expression and formation of foam cells, which lead to endothelial dysfunction (Berliner et al., 2009; Golia et al., 2014), including the release of proinflammatory cytokines, such as interleukin (IL), interferon-γ (IFN-γ), and tumor necrosis factor (TNF; Ait-Oufella et al., 2006; Moriya, 2019). Many autoimmune diseases (e.g., system lupus erythematosus, psoriasis, and rheumatoid arthritis), are found to correlate with increased cardiovascular risk (Kiss et al., 2006; Hak et al., 2009; Vena et al., 2010; Dougados et al., 2014). When anti-inflammatory therapy is applied to systemic lupus erythematosus patients, the mortality of CVD is lower. The mortality is also lower when anti-inflammatory therapy is given to patients with psoriasis (Leonardi et al., 2012; Papp et al., 2012) and rheumatoid arthritis (Liuzzo et al., 1999; Pasceri and Yeh, 1999). Moreover, inflammatory responses, including the monocyte-macrophages, neutrophils, T-cell subsets, and oxidative stress, also contribute to the initiation and development of heart failure (Tanai and Frantz, 2016).

Substrate metabolism is essential for normal cellular physiological function, carbohydrates (e.g., glucose and lactate), and fatty acids are the general cellular energy substrates (Ussher et al., 2016). The production of ATP in the heart is derived mainly from mitochondrial oxidative phosphorylation (OXPHOS), the others come from glycolysis (Bertero and Maack, 2018). When the cardiac supply cannot satisfy the demand, the heart will shift from one substrate to another. The glucose metabolism produces much more phosphates, but less ATP than lipids, which means that glucose metabolism expends less oxygen compared to fatty acid oxidation (FAO) when synthesizing equivalent ATP (Nagoshi et al., 2011). As is shown in the Randle cycle, the lipid metabolism correlates with glucose metabolism in a competitive manner (Randle et al., 1963; Randle, 1998; Sugden, 2007).

Mechanistically, calcium overload regulates the cardiomyocytes, especially in ischemia/reperfusion. When the blood supply decreases, anaerobic metabolism will be upregulated, but cellular pH and ATP production will decline. Accordingly, the Na^+^/H^+^ exchanger (NHE) excretes hydrogen ions in exchange for sodium ions (Pike et al., 1993; Sanada et al., 2011). Ca^2+^ efflux deficiency, and constriction of the reuptake by the endoplasmic reticulum (ER) due to the lack of ATP, will result in calcium overload. Subsequently, the mitochondrial permeability transition (MPT) pore will open, and the mitochondrial membrane potential will change, further weaken the production of energy. After the blood supply is re-established, a cascade of events will be triggered to aggravate the injury.

Many kinds of cell death were found to be engaged in CVDs. Kuwana et al. (2002) provide compelling evidence that the permeabilization of the OMM is involved in apoptosis. When Ca^2+^ gets access into the mitochondria, and opens the mitochondrial permeability transition pore (mPTP), water flows into the mitochondria, causing it to swell and undergo necrosis (Baines et al., 2005). Matsui et al. (2007) reported that autophagy was mediated by AMP-activated protein kinase (AMPK)-dependent pathway in the heart during ischemia/reperfusion injury. Kanamori et al. (2011) verified that autophagy can protect cardiomyocytes from death when suffering from ischemia.

Ohara et al. (1993) first pictured the hallmark of oxidative stress in CVDs in a hypercholesterolemia model. The redox crosstalk contributes to many diseases, such as atherosclerosis. Endothelial dysfunction initiates the process of atherosclerosis. Oxidized LDL (oxLDL) leads to the release bioactive phospholipids that can activate ECs and promote the pathogenesis of atherosclerosis (Hansson et al., 2006). Judkins et al. (2010) discovered the elevated expression of NOX2, an isoform of NADPH oxidase, in ECs and macrophages of lipoprotein deficient ApoE-/- mice, which leads to the formation of atherosclerotic lesions and increased aortic superoxide production. Two studies led by Nishida et al. (2000, 2002) indicate that activation of G-protein coupled receptors (GPCR) can generate ROS. Experiments on neonatal rat ventricular myocytes verified the function of ROS in activating hypertrophic growth signaling via G-proteins (Dai et al., 2011). In hypertension, ROS elevate the concentration of intracellular Ca^2+^ as a second messenger, causing vasoconstriction (Brito et al., 2015). The NOX signaling pathway is important in vascular processes, and lack of NO (nitric oxide), but increased oxidative stress, can be observed in hypertension (Touyz, 2004). Dudley et al. (2005) found increased oxidative stress and O^2⋅–^ production relating to NADPH oxidase in an atrial fibrillation model. Aforementioned articles show evidence suggesting the function of oxidative stress in various kinds of CVDs, but further research on the mechanisms of oxidative stress may produce some unexpected breakthroughs.

### Oxidative Stress-Induced Ferroptosis and CVDs

Cardiomyocytes account for approximately 75% of the heart’s volume, and are rich in mitochondria. They are the main source of cardiac energy metabolism, and are the main site for the production of reactive oxidative species (ROS). Different kinds of cell death, including apoptosis, necrosis, pyroptosis, and ferroptosis, have been shown to be involved in the pathophysiologic process of various CVDs. Studies that describe the roles of ferroptosis in CVDs are listed in Table 1.

**TABLE 1:** Existing studies suggesting the roles of ferroptosis in cardiovascular diseases.

**References**	**Year**	**Models**	**Findings**	**Pathways**
Li et al., 2020	2020	DM model were injected with streptozotocin in the tail vein	Inhibited ferroptosis could alleviate diabetes myocardial IRI	ATF4-CHOP pathway
				ERS pathway
		I/R model was made by ligation of LAD		
Chen et al., 2019	2019	Aortic banding (AB) group and sham-operated (SO) group	Increased TLR4 and NOX4 in HF; activated autophagy and increased ferroptosis	TLR4/NADPH oxidase 4 pathway
		TLR4-siRNAs group and NOX4-siRNAs group		
Feng et al., 2019	2019	Sham hearts, excised hearts in perfusion with KH buffer+LIP-1, or KH buffer+vehicle	Decreased infarct size, increased mitochondrial function	VDAC1
				GPX4
			Lip-1 protected heart from I/R injury	
Li et al., 2019	2019	Non-transplant-related myocardial IRI with vehicle or Fer-1. WT, TLR4-, CD14-, and Trif-deficient hearts	Inhibited ferroptosis and targeted the TLR4/Trif/type I IFN pathway improved IRI and inflammation after heart transplant	TLR4/Trif/type I IFN pathway
Song et al., 2021	2021	AMI models with infusion of PBS or exosomes	Decreased AMI mice myocardial injury through inhibiting ferroptosis	miR-23a-3p
				DMT1
Tang L. J. et al., 2021	2021	Rat model of myocardial ischemia or IRI	Ferroptosis mainly occurred in the phase of myocardial reperfusion but not ischemia	ACSL4, iron, malondialdehyde, and GPX4
Wang J. Y. et al., 2020	2020	A TAC mice model to establish Chronic Heart Failure	MiR-351 can decreased the level of MLK3	The JNK/p53 signaling pathway
Wang C. Y. et al., 2020	2020	Cecal ligation and puncture (CLP) operation. Control (ctrl), CLP, CLP + Dex, and CLP + Dex + YOH groups	Decreased sepsis-induced myocardial ferroptosis	HO-1, iron
				GPX4
Tadokoro et al., 2020	2020	Doxorubicin-induced cardiomyopathy (DIC) model in GPx4 Tg mice and GPx4 hetKO mice	Decreased GPX4 and increased ferroptosis in mitochondria	GPX4
Nemade et al., 2018	2018	Purified human iCell cardiomyocytes which are derived from hiPSCs treated with/without etoposide	The inhibitor of ferroptosis and apoptosis attenuated the heart injury caused by ETP	p53-mediated ferroptosis pathway
Park et al., 2019	2019	Myocardial infarction mouse model	Downregulation of GPX4 in MI advanced ferroptosis in cardiomyocytes	Glutathione, ROS, and GPX4

*DM, diabetes mellitus; LAD, left anterior descending branch; I/R, ischemia/reperfusion; IRI, ischemia reperfusion injury; AB, aortic banding; SO, sham-operated; AMI, acute myocardial infarction; CLP, Cecal ligation and puncture; DIC, Doxorubicin-induced cardiomyopathy; HF, heart failure; Dex, Dexmedetomidine.*

With the development of international research on ferroptosis, various types of iron death inducers and inhibitors were invented, but the specific mechanisms remain unknown. Myocardium iron overload is detected in mice I/R model, and the treatment of ferroptosis inhibitors can greatly improve cardiac function after I/R (Bulluck et al., 2016; Fang et al., 2019). Proteomic studies found that the down-regulation of myocardial GPX4 expression was detected in the early-stage (1 day) and mid-term (1 week) of myocardial infarction in mice, and inhibition of GPX4 expression or function in an *in vitro* model can significantly increase ferroptosis of myocardial cells (Park et al., 2019). Park et al. (2019) revealed that ROS and GPX4 is downregulated in the progression of MI regarding the involvement of the glutathione metabolic pathway. Li et al. found that severe myocardial damage is observed in DM rat with I/R and cell in high-glucose reoxygenation. Ferrostatin-1, the inhibitor of ferroptosis, reduces the endoplasmic reticulum stress (ERS) and myocardial injury in diabetes mellitus (DM) rats with I/R, whereas erastin shows the opposite effect (Li et al., 2020). Ferroptosis is thought to contribute in the progression of heart failure. Chen et al. (2019) found that toll-like receptor 4 (TLR4) and NADPH oxidase 4 (NOX4) were up-regulated and differentially expressed genes (DEGs) in myocardium resulting from heart failure. The HF rats with knock-down of TLR4 and NOX4 by lentivirus siRNA were detected with attenuated autophagy and ferroptosis, improved heart function, and decreased death of myocytes. Friedmann Angeli et al. (2014) showed that liprostatin-1 suppresses ferroptosis in human cells. Feng et al. (2019) provided evidence suggesting that Lip-1 reduced the size of MI and preserved the mitochondrial function via the I/R model reperfused with Lip-1, further study suggested that Lip-1 treatment reduced VDAC1 level and oligomerization, increased antioxidant GPX4 protein level and decreased mitochondrial ROS production. Tang et al. concluded that ferroptosis participates in the phase of reperfusion rather than ischemia (Tang L. J. et al., 2021). The addition of ferrostatin-1 leads to reduced size of MI, and it improved systolic function. It is proposed that ferroptosis and the TLR4/Trif/type I IFN signaling pathway initiate the inflammation which is involved in the adhesion of neutrophils and endothelium after cardiac transplantation (Li et al., 2019). Human umbilical cord blood (HUCB) mesenchymal stem cells (MSC)-derived exosomes inhibited ferroptosis, and exhibited cardioprotective effects on myocardial injury of acute myocardial injury mice, which may be related to the reduced divalent metal transporter 1 (DMT1) expression caused by miR-23a-3p (Song et al., 2021). Mixed lineage kinase 3 (MLK3) regulates oxidative stress through the JNK/p53 signaling pathway, inducing ferroptosis in the pathophysiologic process of myocardial fibrosis under pressure overload (Wang J. Y. et al., 2020).

Research conducted by Wang C. Y. et al. (2020) found that dexmedetomidine can promote sepsis-related myocardial ferroptosis and heart injury, acting through the decline of Ho-1 overexpression, iron levels, and GPX4 activity. Doxorubicin (DOX) is a traditional anthracycline chemotherapeutic with dose-dependent cardiac toxicity. In a study conducted by Tadokoro et al., DOX induced ferroptosis via downregulation of GPX4 and lipid peroxidation in mitochondria (Tadokoro et al., 2020). The other anti-cancer drug, etoposide (ETP), also causes cardiotoxicity. Human pluripotent stem cell-derived cardiomyocytes (hPSC-CMs) treated with liproxstatin-1 had increased function after the addition of ETP. The activation of the p53-mediated ferroptosis pathway by ETP is the key toward ETP-induced cardiotoxicity (Nemade et al., 2018). In summary, ferroptosis can be a target for protection against many CVDs, such as autotaxin (ATX), ferritin H, rapamycin, apart from ferroptosis inhibitors, such as ferrostatin-1 and liproxstatin-1 (Baba et al., 2017; Bai et al., 2018; Fang et al., 2019, 2020).

## Epigenetic Regulators of Ferroptosis and Oxidative Stress

### Epigenetic Regulators of Ferroptosis

In 1942, Waddington CH first proposed the name “epigenotype” and used the term “epigenetics” as the branch of biology emphasizing the relation between genes and their products (Waddington, 2012). Owing to the technological advances and new discoveries, the definition of epigenetics has evolved. Nowadays the most common definition is “the study of mitotically and/or meiotically heritable changes in gene function that cannot be explained by changes in DNA sequence” (Bonasio et al., 2010). One hallmark of epigenetics is the fixed nucleotide sequence (Goldberg et al., 2007). It is a new direction for the therapy of some related diseases. Epigenetics is a bridge that links genotype and phenotype. Currently, the epigenetic process can be clarified into DNA methylation, histone modification (including methylation, acetylation, phosphorylation, ubiquitination, and SUMOylation) and RNA-based mechanism [including long non-coding RNAs (lncRNAs) and microRNAs (miRNAs)] (Prasher et al., 2020).

Several studies have investigated the effect of some epigenetic molecules in ferroptosis in recent years. In Jiang et al.’s study, lymphocyte-specific helicase (LSH), a DNA methylation modifier, can interact with WDR76 to inhibit ferroptosis at the transcriptional level. WDR76 induce lipid metabolic gene and ferroptosis related gene expression in DNA methylation and histone modification via LSH and chromatin modification, the process is affected by lipid ROS and iron concentration (Tao et al., 2017). LncRNAs are made up of over 200 nucleotides but the ability to code protein is relatively low. Abnormal expression of LncRNAs have been shown to be associated with tumorigenesis. For example, LncRNAs participate in the pathophysiology of non-small cell lung cancers (NSCLC) by regulating ferroptosis (Wu et al., 2020). LncRNA function analysis showed that the ferroptosis pathway is associated with SLC7A11 which was downregulated in XAV939-treated NCI-H1299 cells, giving a potential therapeutic target for NSCLC (Yu et al., 2019). Different LncRNAs play different roles in ferroptosis. P53RRA activates the p53 pathway and influences gene transcription to promote ferroptosis, whereas LINC0336 decreases iron concentration and lipid ROS by interacting with ELAV-like RNA binding protein 1 (ELAVL1) to inhibit ferroptosis (Mao et al., 2018; Wang M. et al., 2019). Deubiquitinase is encoded by BRCA1-associated protein (BAP1). Several studies have revealed that BAP1 can inhibit ubiquitinated histone 2A (H2Aub) occupancy on the SLC7A11 promoter (Zhang et al., 2018). Experiments confirm that the downregulated SLC7A11 leads to cystine starvation and GSH depletion to block ferroptosis (Fan et al., 2018). Monoubiquitination of histone H2B on lysine 120 (H2Bub1) promotes the expression of SLC7A11 and regulates many metabolic-related genes, but the p53-USP7-H2Bub1 axis regulates ferroptosis (Wang Y. F. et al., 2019). Selenium-induced selenome gene augmentation can inhibit ferroptosis and protect neuronal cells at the epigenetic level (Alim et al., 2019). Research shows low DNA methylation and elevated levels of H3K4me3 and H3K27ac upstream of GPX4, indicating that high levels of GPX4 may be related with epigenetic regulation (Zhang et al., 2020). Wang et al. reported that overexpression of KDM3B (a histone H3 lysine 9 demethylase) led to decreased histone H3 lysine 9 methylation, but increased the expression of SLC7A11 with the transcription factor, ATF4 (Wang Y. S. et al., 2020).

### Epigenetic Regulators of Oxidative Stress Response

There are also some epigenetic factors that affect oxidative stress response, but have not yet been proven to be directly related to ferroptosis, owing to the close contact between ferroptosis and oxidative stress, a brief introduction is made here. Experimental studies exploring epigenetic regulators of the oxidative stress response are shown in Table 2.

**TABLE 2:** Experiments on the epigenetic regulators of oxidative stress response.

**References**	**Year**	**Models**	**Findings**	**Pathways**
Xiao et al., 2019	2019	Animals were conducted with apolipoprotein E-deficient (apoE^–/–^) and heterozygous SAHH knockout (SAHH^+/–^) mice	Inhibition of SAHH led to decrease SAH levels in plasma, increase oxidative stress and endothelial dysfunction	p66shc-mediated pathway
Ota et al., 2010	2010	HUVECs were pretreated with vehicle, atorvastatin, pravastatin, or pitavastatin diluted in EGM-2 medium for 1 day	Decreased oxidative stress-induced endothelial senescence	The Akt Pathway
Ota et al., 2008	2008	Proliferating HUVECs exposed for 24 h to the indicated concentrations of sirtinol (Calbiochem) or nicotinamide (NAM, Wako Chemical Industries) diluted in medium.	Decreased oxidative stress-induced premature senescence	SIRT1
Hu et al., 2019	2019	NRVFs and rat aortic smooth muscle cells were equilibrated in corresponding medium with 0.1% FBS for 24 h prior to incubation with DMSO, TMP195, or AI-1 for 48 h	HDAC5 inhibition stimulated cardiac NRF2 activity by triggering oxidative stress and HDAC5 catalytic activity reduced cardiomyocyte oxidative stress	NRF2
Costantino et al., 2018	2018	Diabetes was induced in 4-month-old male C57/B6 mice by a single high dose of streptozotocin. An equal volume of citrate buffer was administered in control animals	P66Shc upregulated and induced oxidative stress in the diabetic heart. In vivo gene silencing of p66Shc rescued diabetes-induced myocardial dysfunction	P66shc
Xu et al., 2017	2017	The specific HDAC3 inhibitor RGFP966 and pan-HDAC inhibitor valproic acid were subcutaneously injected into the mice every other day for 3 months	RGFP966 prevented diabetes-induced cardiac dysfunction, inhibited diabetes-induced oxidative stress and inflammation in the mouse	DUSP5-ERK1/2 pathway
Hussain et al., 2020	2020	Diabetes was induced by streptozotocin and control group	Decreased JunD mRNA and protein expression in STZ-induced diabetes	JunD

*apoE^–/–^, apolipoprotein E-deficient; SAH, S-adenosylhomocysteine; SAHH, SAH hydrolase; HUVECs, Human umbilical vein endothelial cells; STZ, streptozotocin.*

Many studies regarding epigenetic regulation have been conducted in cancers, mental illnesses, and immune diseases in recent years. Epigenetic regulation also plays a strong part in CVDs. In a study conducted by Xiao et al. (2019), they found that S-adenosylhomocysteine (SAH) levels in plasma were positively correlated with oxidative stress, and were inversely correlated with flow-mediated dilation and methylation of p66shc promoter in CAD (coronary artery disease) patients and normal subjects. Further research indicates that inhibition of SAH hydrolase results in the increased level of SAH and oxidative stress by epigenetic regulation of p66shc expression, leading to the endothelium injury that may accelerate the progression of atherosclerosis (Xiao et al., 2019).

Nitric oxide is a fundamental molecule that can regulate vasodilatation and prevent vascular inflammation (Tsutsui et al., 2006). SIRT1, a class III histone deacetylase involved in the aging of mice fibroblasts, human ECs, and tumor cells (Ota et al., 2006), may be relevant to the production of ROS and oxidative stress (Hwang et al., 2013). Ota et al. conducted a series of studies on the effects of SIRT1 in ECs. The elevated level of NO strengthens the SIRT1 activity and delays endothelial senescence, but the accumulation of oxidative stress and decreased production of NO in aging will lead to SIRT1 inactivation (Ota et al., 2008). Cilostazol, a selective inhibitor of PDE3, protects ECs from ischemic damage by producing NO. Ota and his colleagues observed cells treated with H_2_O_2_ or Cilostazol, and evaluated the expression of senescence-associated beta-galactosidase assay (SA-betagal). They found that cilostazol increased phosphorylation of Akt at Ser473, as well as eNOS at Ser1177, but the phosphorylation increased SIRT1 expression in a dose-dependent manner (Ota et al., 2008). A similar experiment was conducted by Ota et al. to determine the mechanisms underlying the vascular protective effects of statins. Statins prevent the endothelium from aging by enhancing SIRT1 through the Akt pathway (Ota et al., 2010). Hu et al. found that HDAC5 catalytic activity inhibits cardiomyocyte oxidative stress via NRF2 stimulation. The selective class IIa HDAC inhibitors, TMP195 or TMP269, or shRNA-mediated knockdown of HDAC5 can lead to NRF2-mediated transcription (Hu et al., 2019).

In the diabetic heart, the expression of p66shc increases, but 3-week intensive glycemic control cannot reverse it. Further experiments, which silence the gene of p66shc *in vivo*, lead to the inhibition of ROS and promotion of cardiac function. Upregulation of miR-218 and miR-34a results in changes of the DNMT3b/SIRT1 axis in the diabetic heart, which may be a potential target to cure diabetic cardiomyopathy (Costantino et al., 2018). Another study, conducted by Hussain et al. (2020), found that JunD (a member of AP-1 transcript family) mRNA and protein are shown to have decreased expression in STZ-induced diabetes, which is relevant to oxidative stress, and is regulated by DNA hypermethylation, post-translational modification of histone markers, and translational inhibition by miRNA. Xu et al. treated diabetic mice with HDAC3 inhibitor, RGFP966, and found improved heart dysfunction, hypertrophy, fibrosis, and diminished oxidative stress. Furthermore, increased phosphorylated extracellular signal-regulated kinases (ERK) 1/2 and decreased dual specificity phosphatase 5 (DUSP5) were observed, but RGFP966 can reverse this. Elevated histone H3 acetylation plays an important role DUSP5 gene promoter in diabetic cardiomyopathy (Xu et al., 2017).

## Epigenetic Regulators as Novel Therapeutics

Over the past two decades, mounting efforts have been made to uncover new ways for cardiac repair, such as drug development (e.g., diuretics and ARNI), cardiac devices [e.g., pacemakers and implantable cardiac defibrillators (ICD)], and operations [e.g., electrical defibrillations and transcatheter aortic valve replacement (TAVR)]. In addition, the prognosis of CVD is not satisfied, and further investigation exploring fundamental mechanisms of impaired cardiomyocytes is needed. Epigenetic regulators provide a potential kind of therapy to treat CVDs, which will lay the foundation for individualized medical care.

Iron metabolism homeostasis is strictly regulated by multiple genes, including divalent metal transport-1 (DMT1), TFR1, TFR2, ferroportin (FPN), hepcidin (HAMP), hemojuvelin (HJV), and Ferritin H (Duan et al., 2020). Moreover, epigenetic regulators, such as DNA methylation, histone acetylation, and microRNA participate in iron metabolism homeostasis.

The therapies of CVDs targeting epigenetics are relatively rare. As mentioned above, SIRT1 expression has positive effects in many diseases, including cancer, CVDs, chronic obstructive pulmonary disease (COPD), and type 2 diabetes (Satoh et al., 2011). Resveratrol, a SIRT1 activator, has been suggested to improve heart function via vasodilation, antioxidant activity, and platelet aggregation (Baur and Sinclair, 2006). Resveratrol regulate the vasorelaxant activity through Ca^2+^-activated K+ channels (Li et al., 2000) and NO signaling in the endothelium (Orallo et al., 2002). Das et al. suggested resveratrol upregulates both endothelial and inducible NO synthase (eNOS and iNOS) (Das et al., 2005). Cilostazol protects ECs from ischemic injury by increasing SIRT1-dependent eNOS phosphorylation, producing substantial NO, and the inhibition of SIRT1 leads to inactivation of cilostazol on premature senescence (Ota et al., 2008). Currently, cilostazol is a common clinical drug aiming at ameliorating damage from ischemic injury. A novel SIRT activator, 1,4-dihydropyridine derivatives (DHPs), shows enhanced mitochondrial activity involving PGC-1α (Mai et al., 2009).

Noncoding RNAs are reportedly a potential target for therapeutics in CVDs (Lucas et al., 2018). In acute myocardial infarction, several families of miRNAs kick in, miR-34 can promote telomere erosion, and can regulate the target gene PNUTS (Bernardo et al., 2012; Boon et al., 2013), miR-24 target sirtuin 1, and can regulate EC apoptosis (Fiedler et al., 2011). Inhibition of miR-25 promotes heart function pertaining to the calcium uptake pump, SERCA2a (sarco/ER Ca^2+^-ATPase 2a) (Wahlquist et al., 2014). The related therapies include antisense oligonucleotides, siRNAs, antagomiRNAs, and antimiRNA application.

Yang K. C. et al. (2014) described the myocardial RNA sequence, suggested that the expression profiles of lncRNAs, but not mRNAs or miRNAs, can predict the different pathology of failing heart, indicating the important role of lncRNAs in CVDs. The lncRNA Mhrt (myosin heavy chain-associated RNA transcript) was found repressed under pathological stress condition such as pressure overload-induced hypertrophy and showed cardioprotective effects when restore the physiological concentration (Han et al., 2014). Piccoli et al. (2017) provided evidence suggesting that Inhibition of lncRNA Meg3, which is rich in cardiac fibroblasts, can prevent cardiac fibrosis and diastolic dysfunction. Mutations of the lncRNA H19 have been found related to coronary artery disease (Gao W. et al., 2015). The expression of H19 is highly reduced in atherosclerotic plaques or vascular injury, indicating the important role in cardiovascular system (Kim et al., 1994; Han et al., 1996). LncRNA therapeutics are also promising, in addition to inhibit lncRNA by antimiRs, the function of lncRNA can be blocked by shRNAs including siRNAs, modifed ASOs (antisense oligonucleotides), and gapmers (Lucas et al., 2018).

A growing number of experiments show evidence suggesting the involvement of epigenetics in cancer, CVDs, and metabolic diseases, which provides new ideas on the therapy of refractory diseases. Precision medicine and personalized therapy is the trend, as the development of medicine, genomics, and epigenetics will be the most important tools of the new generation of doctors.

## Conclusion

Ferroptosis is a novel programmed cell death involving inhibition of enzyme GPX4 and lipid hydroperoxides, which was first widely studied in oncology. Oxidative stress-induced ferroptosis have been found to be extensively involved in the biogenesis and development of CVDs, and the inducers, the inhibitors, and the pathways of ferroptosis have been widely explored. Nowadays, the understanding of the role of epigenetics in ferroptosis have greatly increased. However, epigenetic mechanisms, such as lncRNAs, histone monoubiquitination, and DNA methylation are showed to engage in the ferroptosis process involved with CVDs.

Research on epigenetic drugs for CVDs has made great achievements, such as resveratrol, cilostazol, and miRNA family, which reveal the potential of epigenetic therapy for CVDs. However, the current epigenetic molecular mechanism of ferroptosis and the study of cardiac ferroptosis still need to be studied in depth. Thorough research in both basic research and clinical studies, are necessary to fully elucidate the relationship between ferroptosis and epigenetics in CVDs. Some biology approaches including total RNA-sequencing (RNA-seq), single cell RNA-seq, chromatin-immunoprecipitation-sequencing (ChIP-seq), and DNA methylation profiling can help us to further explore the epigenetic regulation with ferroptosis (Xu et al., 2018). And modern biotechnologies such as CRISPR/Cas9, Cre-loxp, proteomics, metabolism omics can comprehensively study the specific mechanisms of epigenetic regulation of ferroptosis for different genes and different stages of iron homeostasis. Hopefully, therapy against epigenetic targets will be promising for treating CVDs in the future.

## Author Contributions

YZ and JLi participated in the design of the review. JLi, YZ, HW, and JLo drafted the manuscript and made the original figures. HC, CL, and YD critically revised the texts and the figures. AS, HC, and YD supervised the research and led the discussion. All authors read and approved the final manuscript.

## Conflict of Interest

The authors declare that the research was conducted in the absence of any commercial or financial relationships that could be construed as a potential conflict of interest.

## Publisher’s Note

All claims expressed in this article are solely those of the authors and do not necessarily represent those of their affiliated organizations, or those of the publisher, the editors and the reviewers. Any product that may be evaluated in this article, or claim that may be made by its manufacturer, is not guaranteed or endorsed by the publisher.
